# Supplementary material for: Cloudy Apple Juice Fermented by Lactobacillus Prevents Obesity via Modulating Gut Microbiota and Protecting Intestinal Tract Health
Source: Nutrients. 2021 Mar 17;13(3):971. doi: 10.3390/nu13030971 (PMC8002442; doi:10.3390/nu13030971)
Supplement: Supplementary file 1 [file nutrients-13-00971-s001.pdf]

Supplementary materials

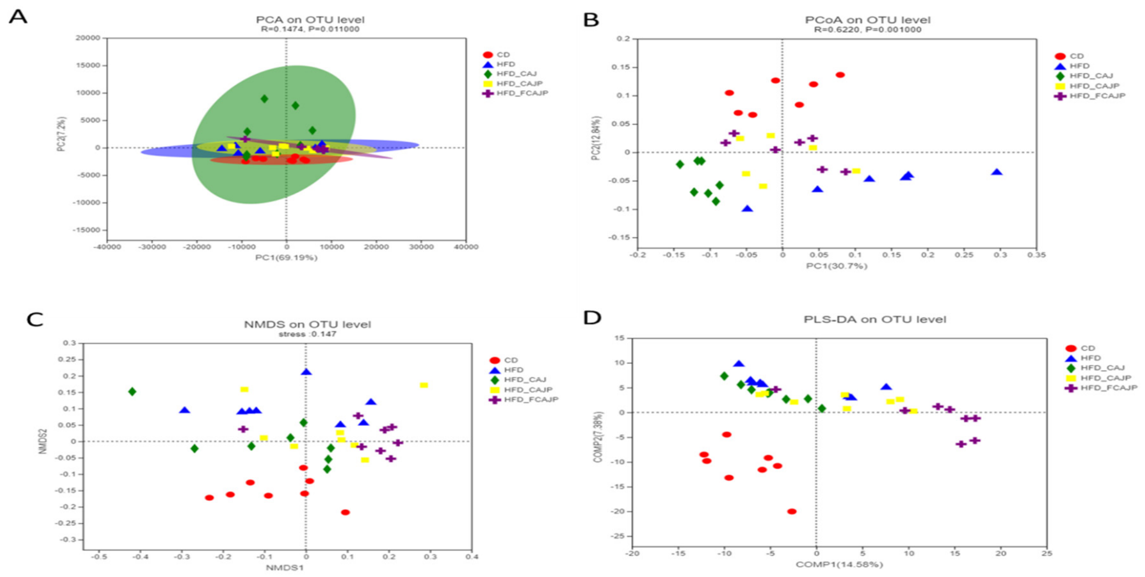

Figure.S1. Responses of the diversity, richness, and structure of the gut microbiota (n=8). **(A)** Principal Component Analysis (PCA) score plot **(B)** PCoA score plot analysis based on Unweighted-unifrac-full-tree; **(C)** Nonmetric multidimensional scaling (NMDS) score plot based on Spearman approx.; **(D)** Partial Least Squares Discriminant Analysis (PLS-DA) score plot. Note: CD: basal diet & Saline; HFD: 45% high-fat diet &Saline; HFD+CAJ: 45% high-fat diet &CAJ; HFD+CAJP: 45% high-fat diet &CAJP; HFD+FCAJP: 45% high-fat diet &FCAJP.

Figure.S2. Five groups comparison chart based on the community abundance at the phylum

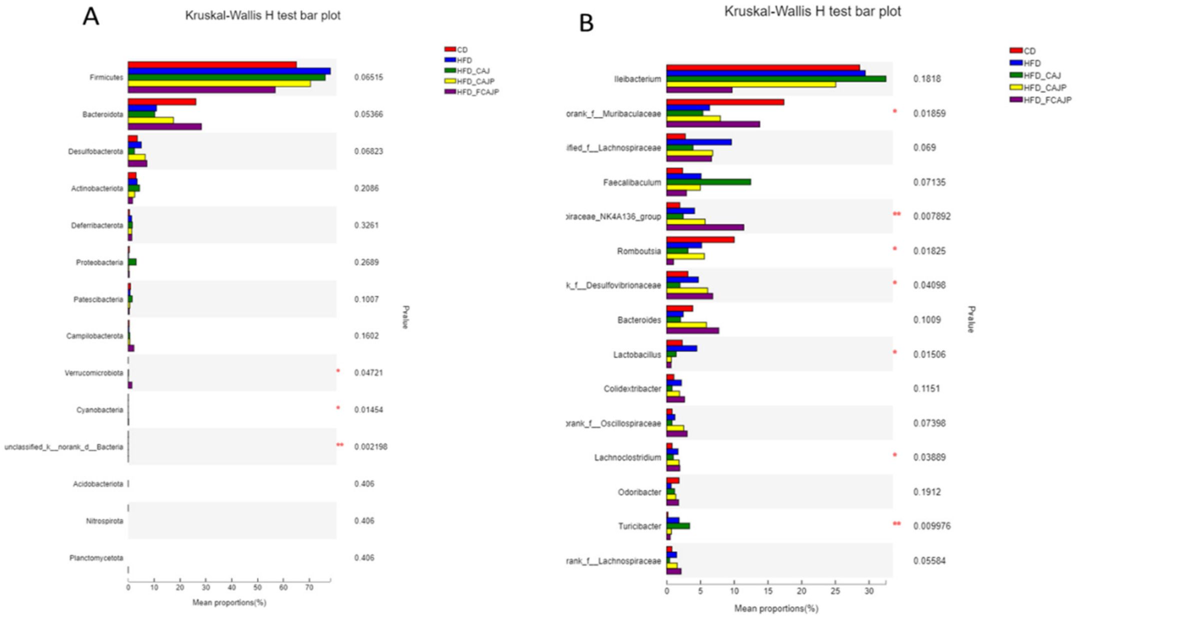

level and genus level (n=8). Note: CD: basal diet & Saline; HFD: 45% high-fat diet &Saline; HFD+CAJ: 45% high-fat diet &CAJ; HFD+CAJP: 45% high-fat diet &CAJP; HFD+FCAJP: 45% high-fat diet &FCAJP. Differences were assessed by Kruskal-Wallis H test and denoted as follows: *$p < 0.05$ and **$p < 0.01$ in a comparison between the two groups.

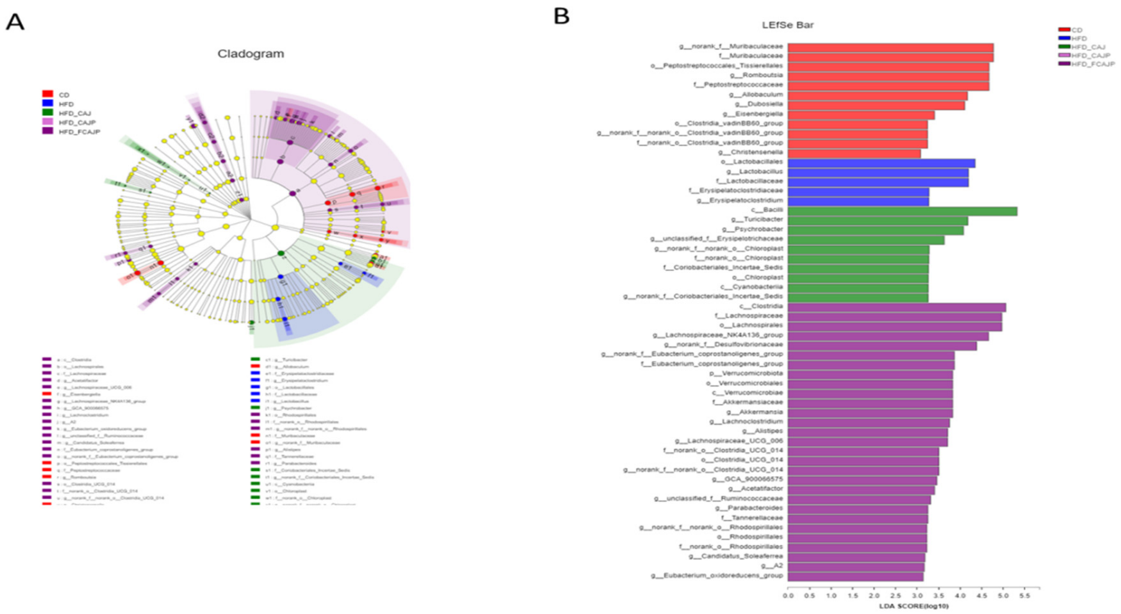

Figure.S3. LEfSe comparison of gut microbiota on groups (n=8). (A) LEfSe multi-level tree map. (B). Distribution histograms of five groups (LDA=3). Note: CD: basal diet & Saline; HFD: 45% high-fat diet &Saline; HFD+CAJ: 45% high-fat diet &CAJ; HFD+CAJP: 45% high-fat diet &CAJP; HFD+FCAJP: 45% high-fat diet &FCAJP.

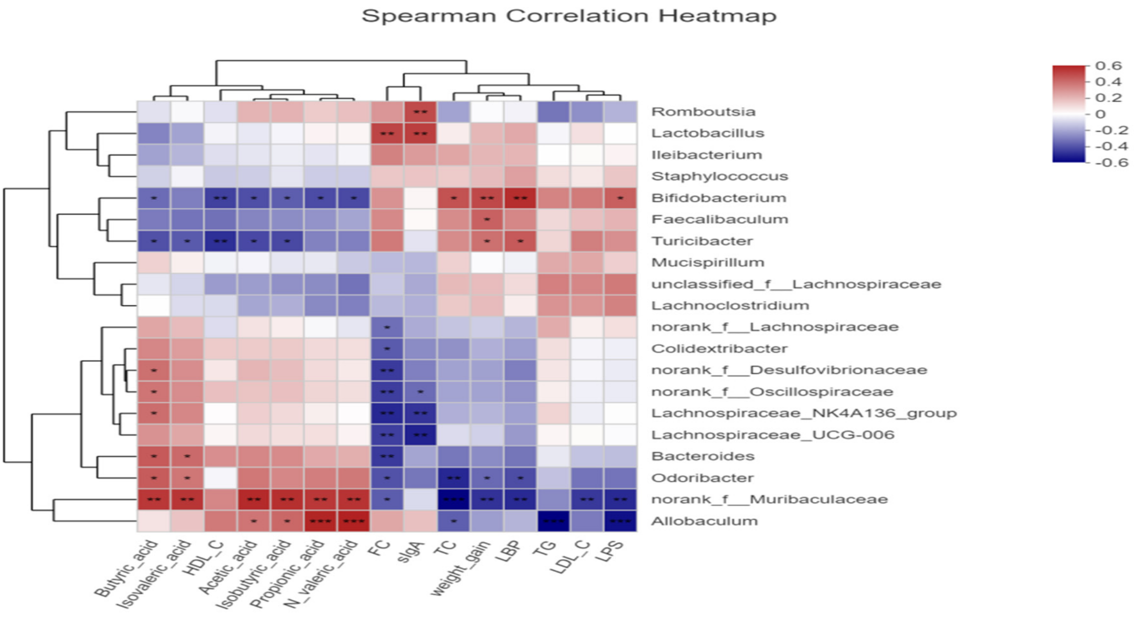

Figure.S4 Spearman's correlation between the relative abundance of gut microbiota and other levels (n=8) *p < 0.05 **p < 0.01 and ***p< 0.001

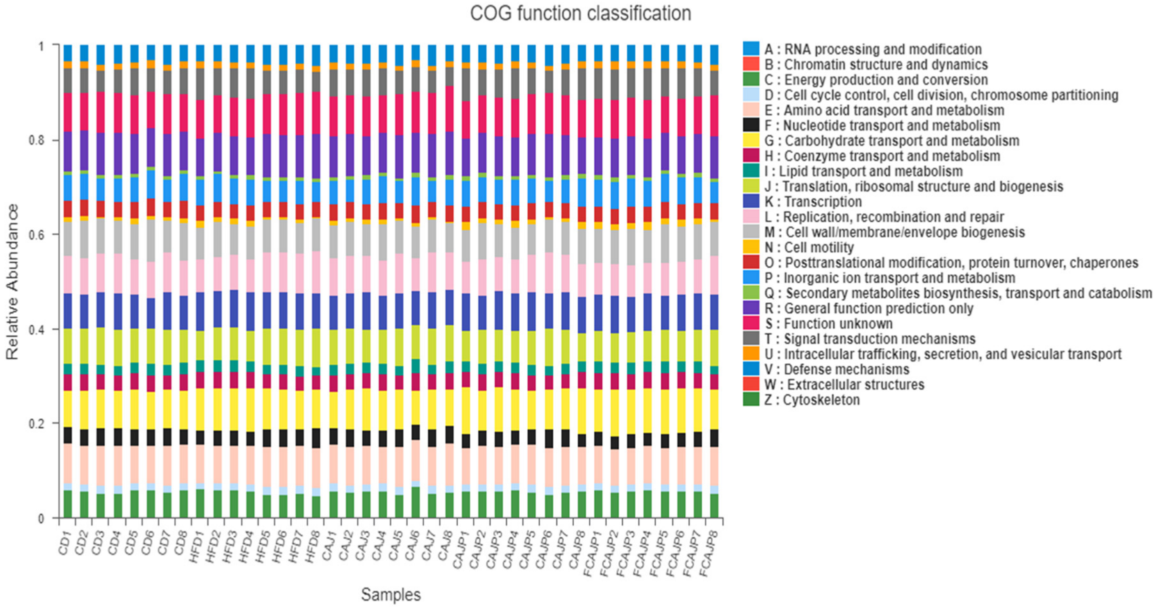

Figure.S5 The abundances of KEGG pathways in the functional prediction by PICRUSt (n=8).

Table S1 The composition of basal diet and 45% high-fat diet

Ingredients (g/kg of diet)	basal diet (g/kg of diet)	45% high-fat diet (g/kg of diet)
Casein	140	175
Corn Starch	465.7	132
Maltodextrin	155	125
Sucrose	100	202
Soybean Oil	40	30
Lard	—	196
Cellulose	50	62
Mineral Mix, M1021	35.0	61
Vitamin Mix, V1010	10	12
L-Cystine	1.8	2
Choline Bitartrate	—	3
TBHQ	0.036	0.045
Choline Chloride	2.5	—
Total	1000	1000

Note:"—" means no the substance

Table2 Daily intake of polyphenols of a mouse in five groups (μg/25g weight of a mouse).

Polyphenols	CD	HFD	HFD+CAJ	HFD+CAJP	HFD+FCAJP
Gallic acid	—	—	0.383±0.007a	0.571±0.004b	1.668±0.013c
Isoferulic acid	—	—	0.060±0.002a	0.574±0.012b	0.749±0.014c
Ellagic acid	—	—	1.032±0.013a	0.935±0.011b	1.064±0.012a
P-hydroxybenzoic acid	—	—	0.039±0.001a	0.053±0.001a	0.089±0.004b
Ferulic acid	—	—	0.204±0.011a	0.164±0.003b	0.169±0.009b
Cinnamic acid	—	—	0.428±0.012a	3.690±0.013b	4.255±0.017c
Chlorogenic acid	—	—	53.996±1.014a	49.583±1.003b	51.840±1.109ab
Proanthocyanidin C1	—	—	0.414±0.021a	0.330±0.014a	0.922±0.005b
Catechins	—	—	0.844±0.015a	1.182±0.012b	1.607±0.005c
Rutin	—	—	0.047±0.002a	1.111±0.019b	0.590±0.002c

Cypridin	—	—	0.551±0.011a	2.210±0.101b	2.428±0.016b
Epicatechin	—	—	11.886±0.304a	12.510±0.207b	9.891±1.308c

Note:"—" means no the substance; in each column mean ± SD value (n = 3 represents the polyphenols in cloudy apple juices were determined in triplicate) bearing different letters differ significantly ($p < 0.05$) between different groups. Note: CD: basal diet & Saline; HFD: 45% high-fat diet &Saline; HFD+CAJ: 45% high-fat diet & CAJ; HFD+CAJP: 45% high-fat diet & CAJP; HFD+ FCAJP: 45% high-fat diet & FCAJP.
